# Complex Fistula-in-Ano Extending From the Anal Canal to Mid-Thigh Treated With Combine Fistulectomy and Video-Assisted Anal Fistula Treatment (VAAFT): A Case Report

**DOI:** 10.7759/cureus.51373

**Published:** 2023-12-31

**Authors:** Zia Ullah, Usaal Sani, Shahzeb Khan, Muhammad Mazher, Saeed Sarwar

**Affiliations:** 1 General Surgery, Khyber Medical College/Khyber Teaching Hospital, Peshawar, PAK

**Keywords:** mri, complex perianal fistulas, fistulectomy, vaaft, fistula in-ano

## Abstract

An anal fistula is abnormal, chronic epithelial-lined communication between the anorectal lumen and the skin of the perineum or buttock. A complex fistula-in-ano is difficult to diagnose and treat; it requires careful approaches because of the high risk of complications and recurrences. We report a case of a 60-year-old male who presented with a discharging sinus on the posteromedial aspect of his left thigh for the past two years. On examination, there were two external openings on the posteromedial aspect of the left thigh, 20 cm away from the anal verge, extending toward the left buttock, with an internal opening on the right side of the anal canal at 11 o'clock in the lithotomy position. The MRI showed a fluid-filled marginally enhancing tract ascending obliquely from the skin of the posterior left thigh, passing through the left gluteus maximus to the bilateral ischioanal fossae. The distal 7 cm tract was excised, as the rest of the tract was deep, passing through the gluteus maximus. Therefore, a video-assisted anal fistula treatment (VAAFT) procedure was done for the remaining tract. The wound was left open for healing by secondary intention. The patient was monitored for six months during which time the wound healed completely.

## Introduction

An anal fistula is abnormal, chronic epithelial-lined communication between the anorectal lumen and the skin of the perineum or buttock [1]. It may be associated with different specific conditions like Crohn's disease, malignancy, tuberculosis, foreign body, and rectal duplications [2]. But, the majority are termed idiopathic, non-specific infections of inter-sphincteric gland infection [3]. A complex fistula-in-ano is difficult to diagnose and treat, it requires careful approaches because of the high risk of complications and recurrences [4]. We describe a combined fistulectomy and video-assisted anal fistula treatment (VAAFT) procedure for complex perianal fistula.

## Case presentation

A 60-year-old man who had undergone coronary artery bypass graft (CABG) and had previously been diagnosed with hypertension and ischemic heart disease presented with a discharging sinus that had been there for the previous two years. The lesion, according to the patient, began as an uncomfortable thigh swelling that was preceded by an insect bite. It subsequently broke open and pus was expelled. Later, it evolved into a sinus that discharges. He also mentioned having perianal discomfort.

**Figure 1:**
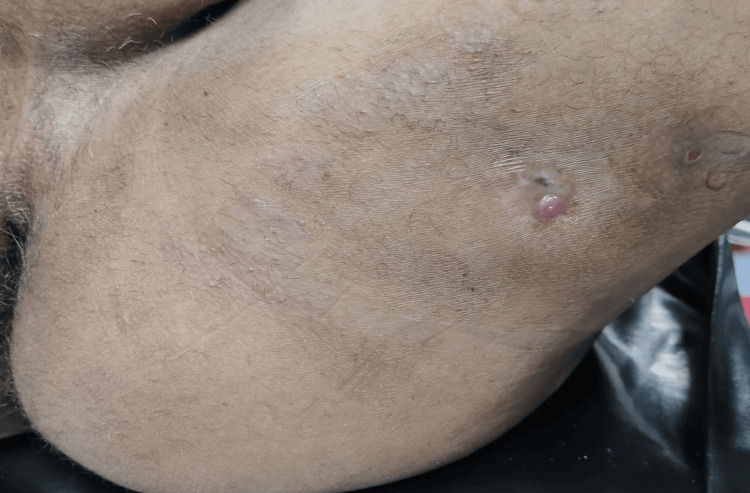
Two external openings on the posteromedial aspect of the left thigh, 20 cm away from the anal verge, extending toward the left buttock

On examination, there were two external openings on the posteromedial aspect of the left thigh, 20 cm away from the anal verge, extending toward the left buttock, with an internal opening on the right side of the anal canal at 11 o clock in the lithotomy position (Figure 1).

**Figure 2:**
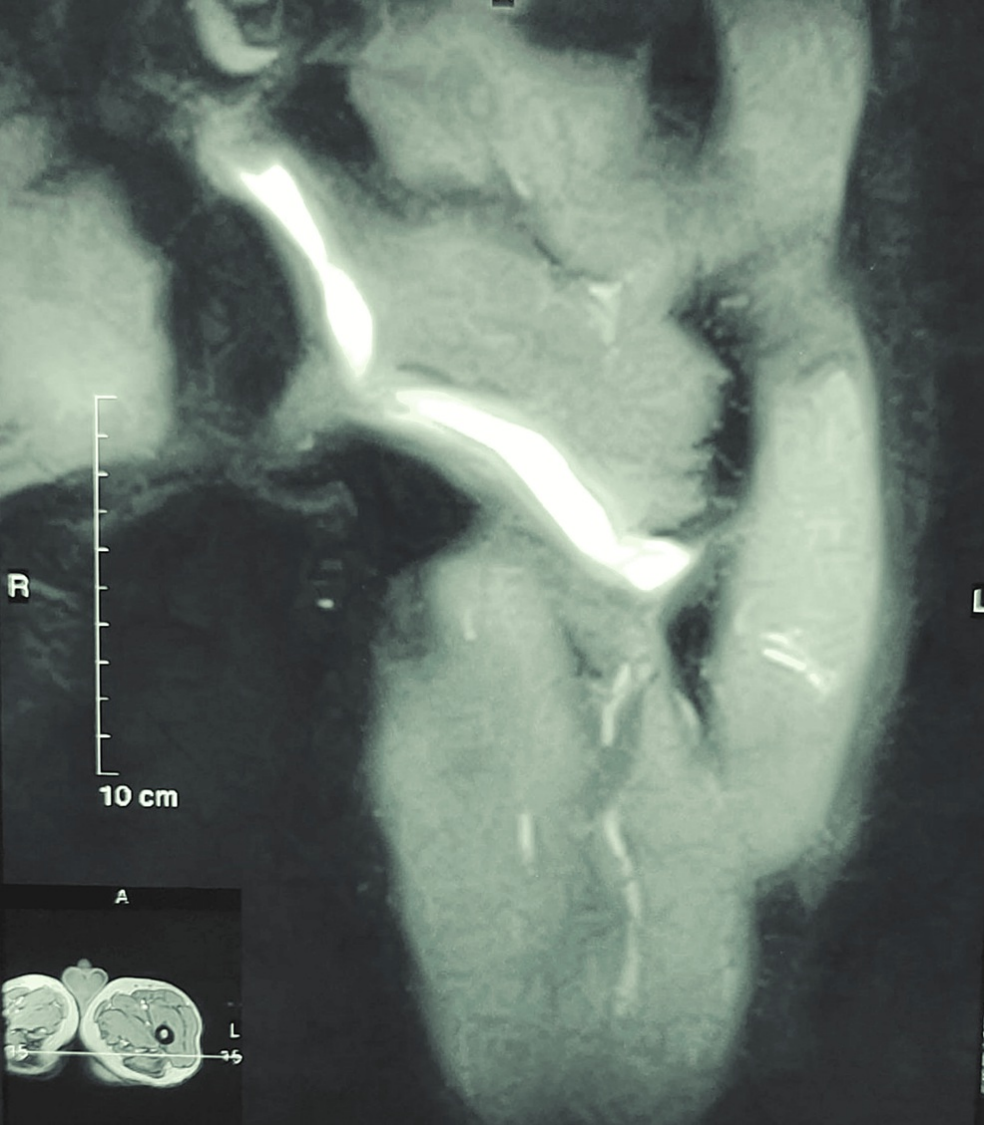
MRI of the left thigh with contrast showing a contrast-filled tract ascending from the skin of the posterior left thigh and ascending to bilateral ischioanal fossae

This finding was consistent with a perianal fistula, which was confirmed by MRI. The MRI showed a fluid-filled marginally enhancing tract ascending obliquely from the skin of the posterior left thigh, passing through the left gluteus maximus to the bilateral ischioanal fossae. There were high signals in the intersphinctric plane of the anal canal at the 1 o’clock position, which is a classic finding in perianal fistulas (Figure 2).

**Figure 3:**
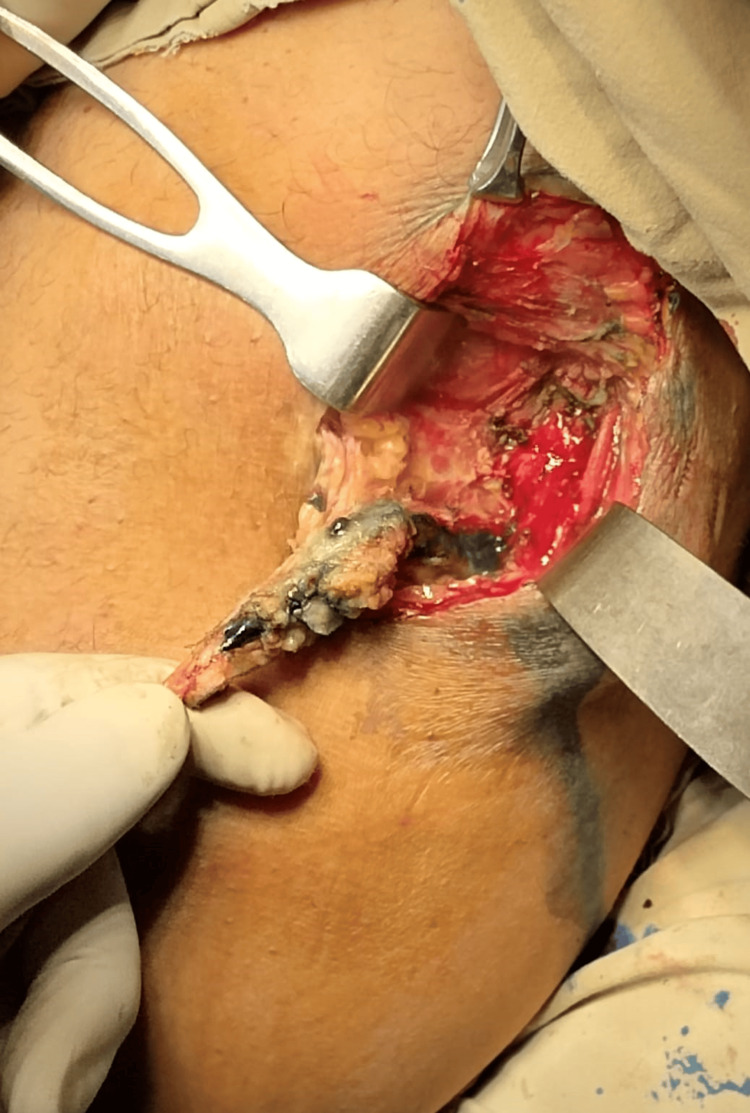
Fistulectomy being performed after identifying the tract with methylene blue dye

**Figure 4:**
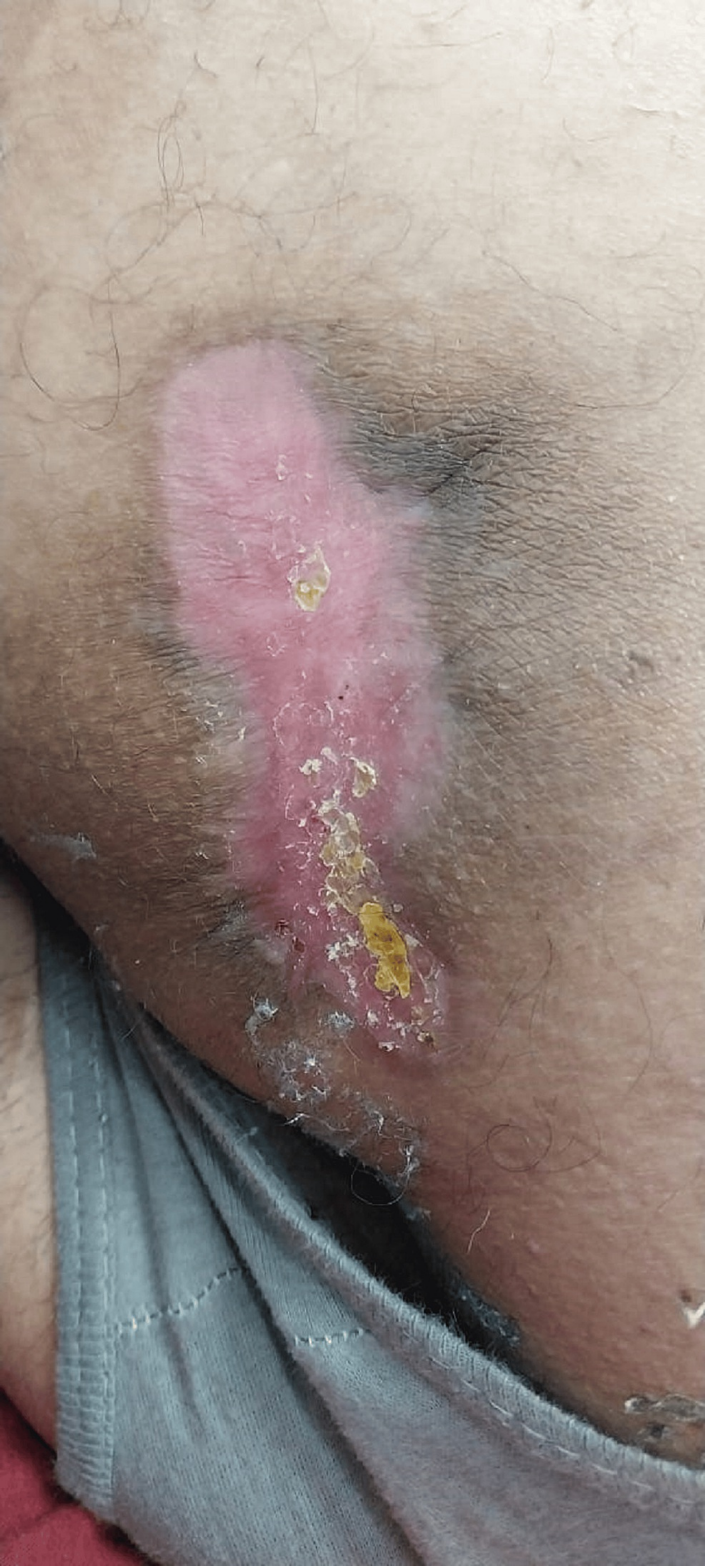
The healed wound after six months

After a thorough workup, the patient was planned for surgery. Under aseptic measures, spinal anesthesia was given, and the patient was positioned in the lithotomy position (both procedures were done in the same position), clean, and draped. Methylene blue was injected into the fistula tract, and the distal 7 cm tract was excised, as the rest of the tract was deep, passing through the gluteus maximus (Figure 3). Therefore, a VAAFT procedure was done for the remaining tract. The wound was left open for healing by secondary intention. The patient was followed for six months. The external wound healed, and there was no discharge after six months (Figure 4).

## Discussion

Due to their complex anatomy and inconsistent presentation, complex anal fistulas are difficult to manage. Fistulectomy is the standard treatment for complex anal fistulas, however, this procedure has a high recurrence and incontinence rate. According to a meta-analysis, the rate of recurrence after surgery for anal fistula ranges from 2.5% to 57.1%, depending on the nature of the fistula [5,6]; moreover, this procedure carries a high risk of other consequences, including slowed wound healing and pain [7]. Because of this, treating complex anal fistulas calls for a customized strategy that considers the anatomy of the fistula, the existence of additional tracts, and any accompanying comorbidities.

The treatment of anal fistulas has been transformed with the emergence of minimally invasive procedures like VAAFT. The fistula tract can be seen and treated using the innovative, minimally invasive VAAFT approach, which lowers the risk of problems brought on by traditional therapies [8].

Anal fistulas can be diagnosed with the help of MRI. It can offer comprehensive details regarding the location, size, and intricacy of the fistula. Additionally, MRI can detect concomitant diseases such as Crohn's disease, cancer, TB, foreign bodies, and rectal duplications [9]. There was no such pathology present in our case, the complicated fistula tract in this instance was identified by the MRI and extended through the gluteus maximus and up to the ischioanal fossae.

VAAFT is a novel, sphincter-saving, and minimally invasive treatment option for complex fistulas [10]. The therapy of difficult perianal fistulas is made possible by the fistulectomy and VAAFT combination outlined in this case report. The VAAFT technique for the remaining tract, followed by the excision of the distal tract, enabled the best possible visualization and treatment of the fistula while lowering the risk of problems brought on by traditional treatments. Complex perianal fistulas can be successfully treated using this strategy, and it may eventually replace other strategies as the preferred method.

## Conclusions

The complex perianal fistula in this case report was successfully treated using a combination of VAAFT and fistulectomy, demonstrating the efficacy of this treatment approach. This case emphasizes the significance of a thorough and multidisciplinary approach to addressing difficult perianal fistulas, with the goal of minimizing the risk of recurrence in addition to relieving symptoms. In complex perianal fistula therapy, the combination of fistulectomy and VAAFT shows encouraging results, providing hope for better patient outcomes and quality of life. To confirm the long-term effectiveness and safety of this strategy, additional studies and long-term follow-up research are required.
